# Orientation of PVDF α and γ crystals in nanolayered films

**DOI:** 10.1007/s00396-015-3542-7

**Published:** 2015-02-28

**Authors:** Kinga Jurczuk, Andrzej Galeski, Matthew Mackey, Anne Hiltner, Eric Baer

**Affiliations:** 1 Department of Polymer Physics, Centre of Molecular and Macromolecular Studies, Polish Academy of Sciences, Sienkiewicza 112, 90-363 Lodz, Poland; 2 Center for Layered Polymeric Systems, Department of Macromolecular Science and Engineering, Case Western Reserve University, Cleveland, OH 44106-7202 USA

**Keywords:** X-ray diffraction, Pole figures, Texture, γ Phase, Poly(vinylidene fluoride)

## Abstract

Wide-angle X-ray scattering in conjunction with pole figure technique was used to study the texture of poly(vinylidene fluoride) (PVDF) α and γ phase crystals in nanolayered polysulfone/poly(vinylidene fluoride) films (PSF/PVDF) produced by layer-multiplying coextrusion. In all as-extruded PSF/PVDF films, the PVDF nanolayers crystallized into the α phase crystals. A large fraction of those crystals was oriented with macromolecular chains perpendicular to the PSF/PVDF interface as evidenced from the (021) pole figures. Further refinement of the texture occurs during isothermal recrystallization at 170 °C in conjunction with transformation of α to γ crystals. The γ crystals orientation was probed with the (004) pole figures showing the *c*-axis of PVDF γ crystals perpendicular to the PSF/PVDF interface. The thinner the PVDF layers the stronger the orientation of γ crystals. It was proven that the X-ray reflections from the (021) planes of α crystals and from the (004) planes of γ crystals are not overlapped with other reflections and can be effectively used for the texture determination of PVDF nanolayers in multilayered PSF/PVDF films.

## Introduction

Poly(vinylidene fluoride) (PVDF) is a partially crystalline linear hydrofluorocarbon polymer exhibiting extraordinary electrical properties, ranging from those of a typical dielectric polymer to those of a versatile ferroelectric material, as a consequence of its crystalline structure and the abundance of polymorphic phases. Four polymorphs, α, β, γ and δ, have been documented so far [1, 2], and the fifth ε form is strongly suggested to exist [3]. A rather strong electric moment in the PVDF monomer unit arises because of a strong electronegativity of fluorine atoms as compared to those of hydrogen and carbon atoms. Thus, each macromolecular chain possesses a dipole moment perpendicular to the polymer chain [4, 5]. If a polymer chain is packed in crystals to form parallel dipoles, the crystal possesses a net dipole moment as in polar β, γ, and δ forms; whereas, in antiparallel chain dipoles alignment, the net dipole moment vanishes as in nonpolar α and ε phases. The most common polymorph of PVDF is the α phase predominantly obtained during crystallization from the melt at moderate or high supercoolings [6, 7]. The α phase is formed also during polymerization, and it is characterized by a trans-gauche-trans-gauche′ (TGTG′) conformation of macromolecular chains. It is nonpolar and does not exhibit ferroelectricity; however, when deformed, it displays a large flexoelectric effect, connected with the strain gradient [8].

The α phase can be transformed into three other polymorphic forms under an action of sufficient mechanical stress, heat, or electrical field. The β phase, usually obtained during mechanical deformation of α spherulites [9–16], is presently the most important polymorph of PVDF used extensively for piezoelectric and pyroelectric applications. An all-trans (TT) molecular conformation of PVDF is responsible for the ferroelectric properties. However, the fluorine atoms are too large to allow a simple all-trans conformation, and they are slightly offset to form a zigzag arrangement along the crystal *c*-axis [17].

The presence of γ phase has been reported in PVDF films crystallized from the dimethyl sulfoxide, dimethyl acetamide, and dimethyl formamide solutions [18, 19] as well as in the samples crystallized at high pressures [20, 21], at high temperatures [6, 7, 22, 23], and after annealing of the α phase crystals [7, 24]. The macromolecular chains of γ phase are in the TTTGTTTG′ conformation and can be considered, regarding the ferroelectric effect, as an intermediate between the α and β phases. When formed during melt crystallization above 160 °C, the γ phase reaches the highest concentration close to 170 °C [25].

The polar δ phase can be formed by poling the antipolar α phase at high electric fields [26, 27]. This form has the same unit cell dimension along *c*-axis and macromolecular chain conformation as the α form, the difference lying in the interchain packing alone.

A fifth crystallographic form is the ε phase containing the TTTGTTTG′ conformation of macromolecular chains similar to the γ phase but in an antipolar arrangement [3].

Mechanical deformation of the γ phase at most temperatures leads to an almost complete transformation to β phase [13, 14], so for many years, oriented films containing γ phase had not been available. However, Mackey et al. [28] showed that isothermal recrystallization of PVDF nanolayers in multilayered PSF/PVDF films at high temperatures causes the phase transition from α to γ phase crystals.

PVDF is used in a wide range of applications due to the ferroelectric properties of β, γ, and δ crystals, which in turn is closely related to the alignment and orientation of those crystals. There are many means of enforcing PVDF crystal alignment and orientation; most of them adopted from known processes designed for commodity polymers.

The methods used to identify the polymorphic phases of PVDF and to determine the orientation of crystals in pure PVDF films or in blends with other polymers such as polyamide 11 (PA 11) [29], poly(methyl methacrylate) (PMMA) [30], polyvinylpyrrolidone (PVP) [31], polycarbonate (PC) [28], and polysulfone (PSF) [28] include mainly X-ray diffraction [1, 9, 14, 29–34], infrared spectroscopy (FTIR) [6, 11, 12, 29, 31, 33, 35–37], Raman scattering [38, 39], and nuclear magnetic resonance (NMR) [36]. Nevertheless, all methods mentioned above enabling identification of PVDF polymorphs, are insufficient to determine fully the preferred orientation of PVDF crystals and the texture of films. In the case of two-dimensional wide-angle X-ray diffraction (WAXS-2D), the information is incomplete because the alignment and orientation of PVDF crystals in the direction along the incident X-ray beam path cannot be elucidated. Full texture determination can be achieved using X-ray pole figures technique [40]. In the literature, there is practically no information about this technique used to examine the texture of PVDF. Only Wang and Cakmak [30] applied WAXS pole figures of (020) plane to examine the orientation of PVDF α phase crystals in injection-molded PVDF and PVDF/PMMA blends.

In this article, we concentrate on the full texture determination of PVDF nanolayers in multilayered PSF/PVDF film systems utilizing X-ray pole figures of reflections from selected crystallographic planes.

## Experimental

### Materials

Semicrystalline poly(vinylidene fluoride) (PVDF) homopolymer Solef® 6010 from Solvay Solexis crystallizing from the melt in the α phase [41] and amorphous polysulfone (PSF), Udel P-3703® obtained from Solvay Advanced Polymers, were chosen as raw materials to produce 12-μm thick PSF/PVDF film systems by layer-multiplying coextrusion at conditions described in details by Mackey et al. [28]. The compositions of all film systems studied in the paper are collected in Table 1. In each layered system, the PVDF nominal layer thickness was varied from 28 to 225 nm. The multilayered PSF/PVDF films were isothermally recrystallized using two oil baths containing silicon oil at recrystallization temperatures of 145 °C and 170 °C, respectively [28].

**Table 1:** The as-extruded and recrystallized multilayered PSF/PVDF film systems

Sample code	Number of layers	Composition (*v*/*v*) PSF/PVDF	PVDF nominal layer thickness (nm)	Recrystallization temperature/time (°C/h)
#1	1	0/100	–	–
#1a	1	0/100	–	145/5
#1b	1	0/100	–	170/96
#2	32	70/30	225	–
#3	32	70/30	225	145/5
#4	256	50/50	47	–
#5	256	50/50	47	170/96
#6	256	70/30	28	–
#7	256	70/30	28	170/96

### X-ray measurements

The overall orientation of crystallographic planes of the samples was determined by means of computer-controlled WAXS system equipped with a pole figure attachment associated with a wide-angle goniometer (DRON 2.0) coupled to a sealed tube source of filtered Cu K_α_ radiation (Phillips), operating at 50 kV and 30 mA. The specimens in the form of sandwiched films (at least 1 × 1 cm) and approximately 0.15 mm thick were assembled with extrusion direction vertical. The WAXS reflection scans of the samples were collected with the step of 0.05°. The X-ray data for pole figure construction were collected for selected reflections. The receiving slits were set to record the integral intensity of the reflection. Experimental X-ray diffraction data were corrected for background scattering, sample absorption, and defocusing of the beam. All pole figures were plotted with the POD program (Los Alamos National Lab, NM). Other details of the experimental procedure are described elsewhere [42].

## Results and discussion

In order to identify and select the X-ray diffraction peaks for further detailed analysis of crystalline structure and texture of our multilayered PSF/PVDF films, we summarized the current knowledge about predicted and observed X-ray reflections from the PVDF crystals. In Tables 2, 3, 4, and 5, the most important known diffraction peaks are collected for the α, β, γ, and δ crystallographic forms of PVDF.

**Table 2:**
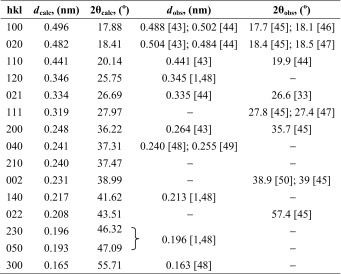
The data for crystallographic planes of α form of PVDF and the observed wide-angle diffraction peaks

*d*_calc_—interplanar distance calculated from the unit cell of α form PVDF crystals (orthorhombic, *a* = 0.496 nm, *b* = 0.964 nm, *c* = 0.462 nm); 2θ_calc_—wide angle of diffraction maximum calculated from the Bragg’s law (*nλ* = 2*d*sinθ), where *λ*, wavelength of the incident X-ray beam has been assumed to 0.15418 nm; *d*_obs_ and 2θ_obs_—interplanar distance and wide angle of diffraction maximum observed in the references [1, 33, 43–50]

**Table 3:**
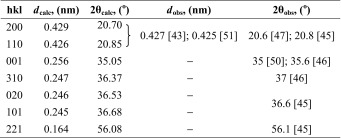
The data for crystallographic planes of β form of PVDF and the observed wide-angle diffraction peaks

*d*_calc_—interplanar distance calculated from the unit cell of β form PVDF crystals (orthorhombic, *a* = 0.858 nm, *b* = 0.491 nm, *c* = 0.256 nm); 2θ_calc_—wide angle of diffraction maximum calculated from the Bragg’s law (*nλ* = 2*d*sinθ), where *λ*, wavelength of the incident X-ray beam, has been assumed to 0.15418 nm; *d*_obs_ and 2θ_obs_—interplanar distance and wide angle of diffraction maximum observed in the references [43, 45–47, 50, 51]

**Table 4:**
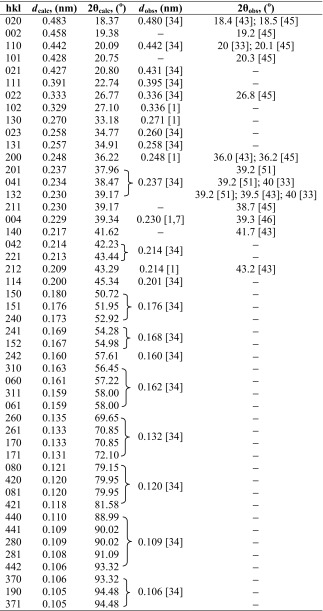
The data for crystallographic planes of γ form of PVDF and the observed wide-angle diffraction peaks

*d*_calc_—interplanar distance calculated from the unit cell of γ form PVDF crystals (monoclinic, *a* = 0.497 nm, *b* = 0.966 nm, *c* = 0.918 nm, β-angle = 92.9°); 2θ_calc_—wide angle of diffraction maximum calculated from the Bragg’s law (*nλ* = 2*d*sinθ), where *λ*, wavelength of the incident X-ray beam, has been assumed to 0.15418 nm; *d*_obs_ and 2θ_obs_—interplanar distance and wide angle of diffraction maximum observed in the references [1, 7, 33, 34, 43, 45, 51]

**Table 5:**
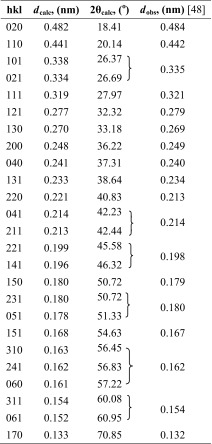
The data for crystallographic planes of δ form of PVDF and the observed wide-angle diffraction peaks

*d*_calc_—interplanar distance calculated from the unit cell of δ form PVDF crystals (orthorhombic, *a* = 0.496 nm, *b* = 0.964 nm, *c* = 0.462 nm); 2θ_calc_—wide angle of diffraction maximum calculated from the Bragg’s law (*nλ* = 2*d*sinθ), where *λ*, wavelength of the incident X-ray beam, has been assumed to 0.15418 nm; *d*_obs_ and 2θ_obs_—interplanar distance and wide angle of diffraction maximum observed in the reference [48]

**Fig. 1:**
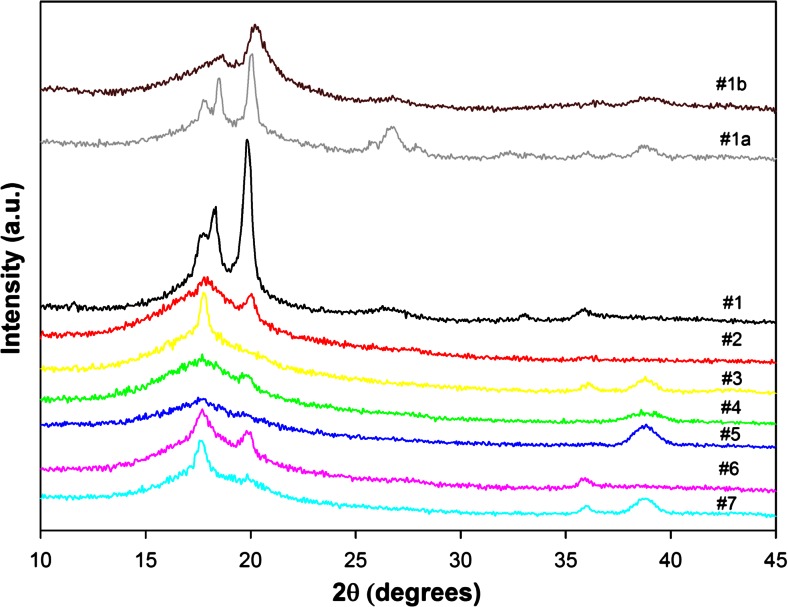
The WAXS diffractograms of as-extruded PVDF control film, PVDF control film annealed at 145 °C for 5 h, PVDF control film annealed at 170 °C for 96 h, and PSF/PVDF multilayered films, obtained in the reflection mode. The curves are numbered according to the sample codes from Table 1. The extrusion direction is vertical. The curves have been shifted for better visualization

Figure 1 presents the WAXS diffractograms of as-extruded PVDF control film, PVDF control film annealed at 145 °C for 5 h, PVDF control film annealed at 170 °C for 96 h, and PSF/PVDF film systems.

For as-extruded 0.15-mm thick PVDF control film (sample #1), five diffraction peaks with maxima at 17.75°, 18.30°, 19.85°, 26.50°, and 35.90° are observed in Fig. 1. Their positions imply that they are reflected from (100), (020), (110), (021), and (200) crystallographic planes of PVDF α phase crystals. No other crystallographic forms were detected in the WAXS scan.

Annealing of PVDF control film at 145 °C for 5 h (sample #1a) causes refining of α crystals, note the intensification of (021) reflection at 26.50° and the appearance of (120), (111), and (002) reflections at 25.55°, 27.80°, and 35.80°, respectively. No other crystallographic forms of PVDF are present in the sample.

Annealing of the PVDF control film at 170 °C for 96 h (sample #1b) drastically changes the crystalline content. The α crystals disappeared and instead of them, the γ crystals appeared. The reflections of (020), (110)/(101), and (004) crystallographic planes of PVDF γ phase crystals are observed at 18.55°, 20.20°, and 39.20°, respectively.

The film #2 contains only 30 vol.% of PVDF in the form of 255-nm thick layers between 70 vol.% of PSF layers. From the X-ray diffraction peaks, it is evident that the PVDF crystals are also of the α phase although the diffraction is of rather low intensity due to lower concentration of PVDF in the film and probably due to a spatial orientation of α crystals.

After isothermal recrystallization at 145 °C of the 225-nm thick PVDF layers in the PSF/PVDF film (sample #3), two diffraction peaks appeared, at 35.85° for (200) plane of α phase and at 39.10°. This last diffraction peak was identified as PVDF γ phase crystal (004) reflex [1, 34, 52]. Other diffraction peaks with (hk0) indices are not well seen in reflection; hence, the crystalline fraction of the sample #3 consists of a combination of α and γ phases, however, oriented.

The film #4 contains 50 vol.% of PVDF in the form of 47-nm layers between 50 vol.% of PSF layers. From the X-ray diffraction peaks in the reflection mode, it is evident that the PVDF crystals are of the α and γ phases, although the diffraction is again of rather low intensity due to a spatial orientation of α and γ crystals. Other diffraction peaks with (hk0) indices are not well seen in the reflection mode due to spatial orientation.

After isothermal recrystallization at 170 °C of the 47-nm thick PVDF layers in the PSF/PVDF film (#5), only one diffraction peak at 39.10° of (004) reflection of γ phase is seen. No sign of α form can be noticed.

In the film #6 with 28-nm thick PVDF layers the diffraction peaks from the α form with diffraction maxima from planes (020), (100), (110), and (200) are observed. No diffraction from (021) of α form and no γ form reflections are noticed.

After isothermal recrystallization at 170 °C of the 28-nm thick PVDF layers in the PSF/PVDF film (sample #7), the diffraction peaks at 17.65°, 35.95°, and 39.10° are seen which are identified as the (100) of α form overlapped with the (020) of γ form, the (200) of α form, and the (004) of γ phase, respectively. Hence, the coexistence of α and γ forms can be noticed.

In thinner PVDF layers, the α phase crystals grow with structural defects and with orientation controlled by the PSF/PVDF interface. In the cases of films #2, #4, and #6 with PVDF nanolayers, the peak from (004) plane of γ phase becomes narrower and more intense when the samples were isothermally recrystallized at 145 or 170 °C. This (004) peak is isolated from other diffraction peaks and we postulate that it can be used for clear and precise determination of *c*-axis orientation of γ crystals. Similar role for the orientation of α crystals can be served by (021) reflection which is also isolated from other diffraction peaks. This crystallographic plane is tilted by 46.22° with respect to *c*-axis of α crystals. It is then postulated that the orientation of α and γ crystals can be precisely determined from pole figures of (021) and (004) planes, respectively.

**Fig. 2:**
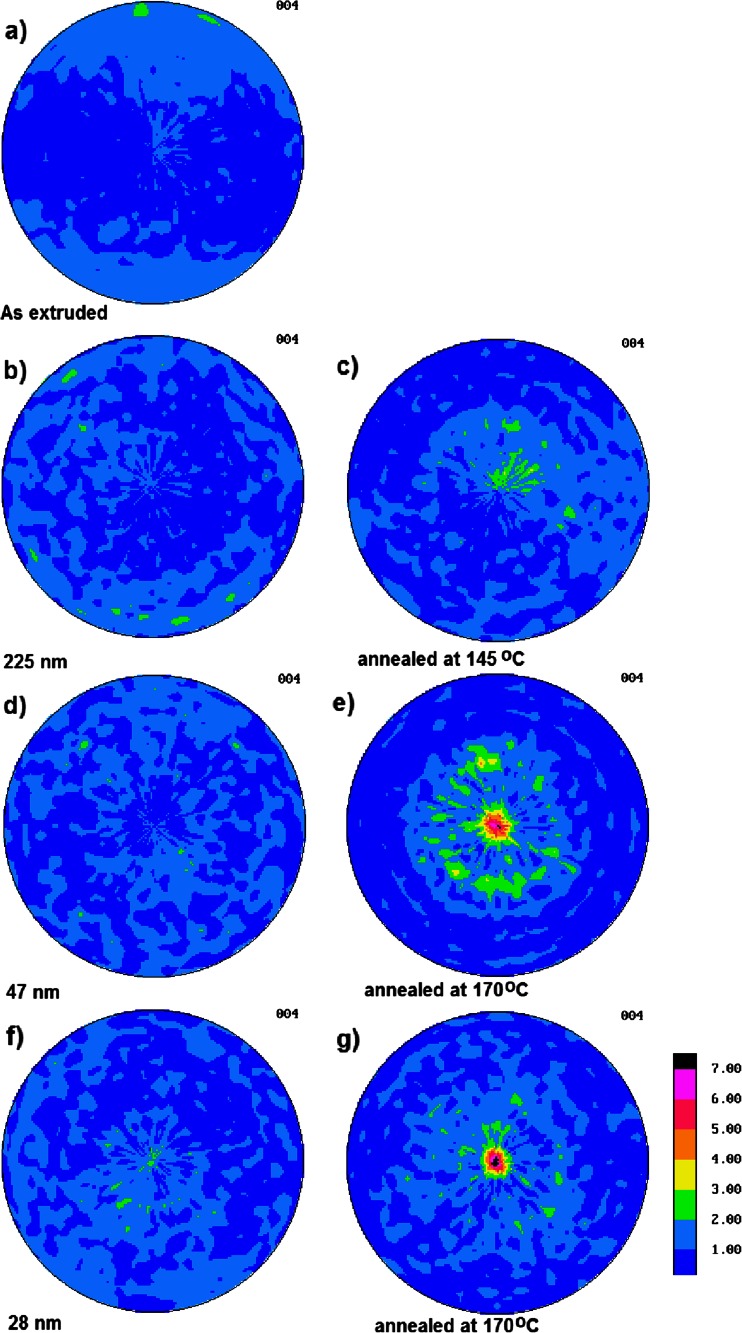
The pole figures of normals to the (004) planes of PVDF γ crystals: **a** as-extruded PVDF control film, **b** PSF/PVDF film with nominal PVDF layer thickness of 225 nm, **c** PSF/PVDF film with nominal PVDF layer thickness of 255 nm isothermally recrystallized at 145 °C, **d** PSF/PVDF film with nominal PVDF layer thickness of 47 nm, **e** PSF/PVDF film with nominal PVDF layer thickness of 47 nm isothermally recrystallized at 170 °C, **f** PSF/PVDF film with nominal PVDF layer thickness of 28 nm, and **g** PSF/PVDF film with nominal PVDF layer thickness of 28 nm isothermally recrystallized at 170 °C. The extrusion direction is *vertical* and the transverse direction is *horizontal*. The normal direction is the *center of pole figures*

The orientation of PVDF chains in the crystals was examined using X-ray pole figures. The pole figures of normals to the (004) plane for all multilayered PSF/PVDF film systems are collected in Fig. 2.

From the pole figure in Fig. 2a, it is seen that there is no preferred orientation of that small amount of γ phase crystals present in the PVDF control film (sample #1), except for a slight orientation due to the extrusion process. The non-annealed film #2 with 255-nm thick PVDF layers also does not show any orientation of γ crystals (Fig. 2b). A brief estimation on the basis of WAXS 2θ diffractogram indicated that the content of γ crystals is low in that sample (see Fig. 1). Annealing at 145 °C of that film produced some small amount of γ crystals (sample #3); however, they are only little oriented with *c*-axis (normal to (004) planes) perpendicular to the film surface, as it can be judged from Fig. 2c. Non-annealed film #4 with 47-nm thick PVDF layers also contain only small amount of γ crystals, and they are unoriented as it can be deduced from the (004) pole figure in Fig. 2d. In contrast, a strong texture of (004) planes is detected in Fig. 2e for film #5 with 47-nm thick PVDF layers after annealing at 170 °C for 96 h. Non-annealed film #6 with 28-nm thick PVDF layers also contained very little amount of γ crystals (Fig. 2f). After isothermal recrystallization of that film at 170 °C for 96 h (sample #7), most of α crystals were transformed to γ crystals showing very strong texture with most of normals to the (004) planes being perpendicular to the film surface (Fig. 2g). PVDF crystals are laying flat to the film plane exactly indicating the suggested alignment of γ phase crystals as showed by Mackey et al. [28]. At higher recrystallization temperature, i.e., at 170 °C PVDF nanolayers recrystallized as in-plane γ phase crystals.

The above data on the orientation of PVDF γ crystals in nanolayers, especially that crystals in thinner layers are more perfectly oriented with *c*-axes perpendicular to the film surface, led to the conclusion that the interface between PSF and PVDF plays an important role in the orientation of γ crystals. However, it is not clear whether the martensitic transformation of α to γ crystals occurs via macromolecular chains reorientation or the chains were already oriented perpendicular to the interfaces in α crystals while the transformation relies on a conformation change from TGTG′TGTG′ to TTTGTTTG′. The answer can be found by investigating the α crystal texture of non-annealed samples.

**Fig. 3:**
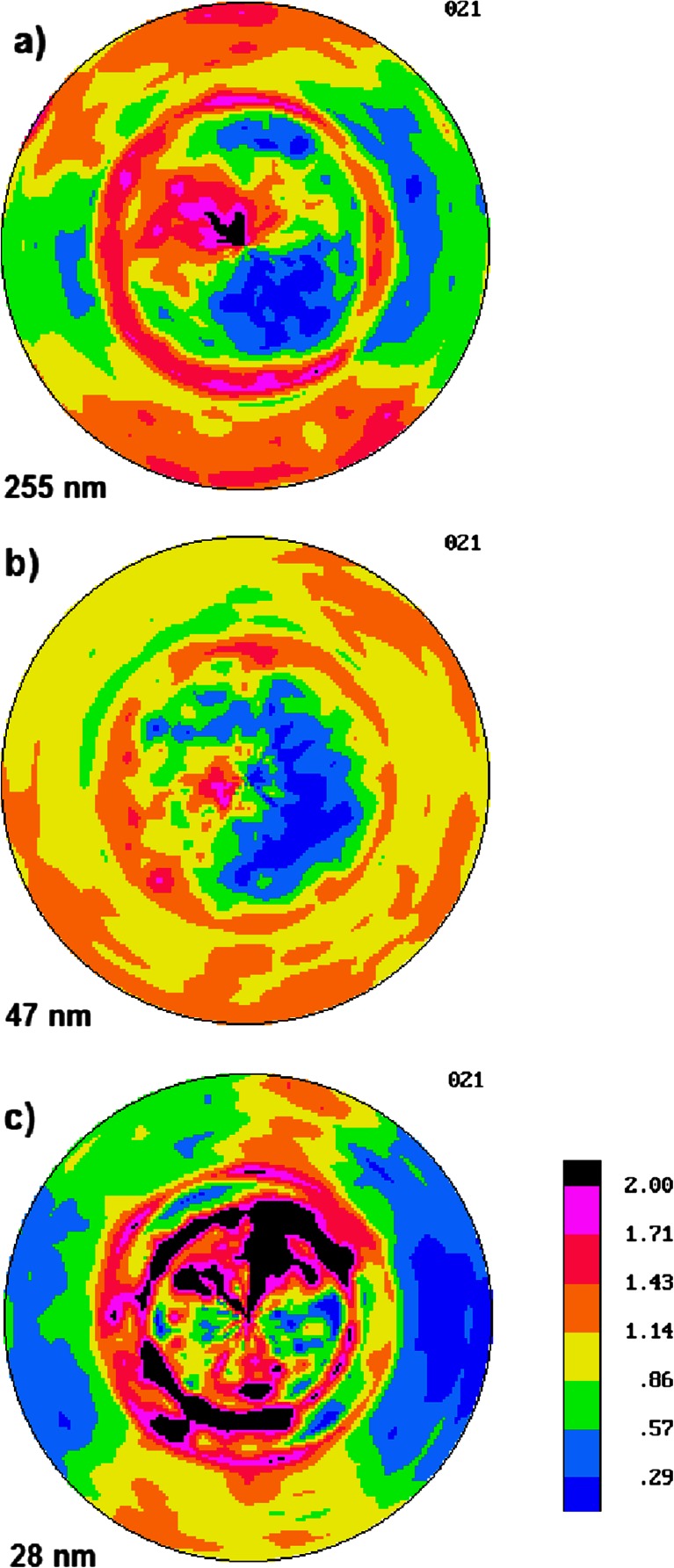
The pole figures of normals to the (021) planes of α crystals in non-annealed multilayered PSF/PVDF films with PVDF nominal layer thickness of **a** 255 nm, **b** 47 nm, and **c** 28 nm. The extrusion direction is *vertical* and the transverse direction is *horizontal*. The normal direction is the *center of the pole figure*

The pole figures of normals to the (021) planes of α crystals for non-annealed multilayered PSF/PVDF films (samples #2, #4 and #6) are presented in Fig. 3.

It is evident that the (021) planes of α crystals are preferentially oriented at 40–45° with respect to the normal to PSF/PVDF interfaces in all three multilayered PSF/PVDF films. Stronger clustering of the (021) normals is observed for thinner 28-nm PVDF layers. However, the texture in all three samples is not very strong. Apparently, further refinement of chains orientation perpendicular to film surface occurs during isothermal recrystallization at 145 or 170 °C facilitated by interaction of PVDF macromolecules with PSF/PVDF interface.

## Conclusions

The PVDF nanolayers in all as-extruded PSF/PVDF films crystallized into the α phase structure. After isothermal recrystallization at 170 °C, α phase crystals in PVDF layers transformed into γ phase crystals.

**Fig. 4:**
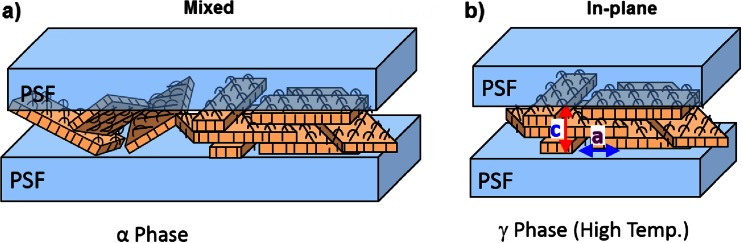
Schematics of the orientation types of PVDF crystals: **a** mixed orientation of α crystals in as-extruded multilayered PSF/PVDF films and **b** in-plane orientation of γ crystals in recrystallized multilayered PSF/PVDF films

The X-ray diffraction in conjunction with pole figures was used to examine the texture of PVDF in multilayered PSF/PVDF films. The (021) planes of α crystals are well suited to use them for the determination of the PVDF crystal texture. There is some orientation of the (021) planes at 40–45° to the PSF/PVDF interface in all as-extruded multilayered films. For γ crystals, the (004) planes may be used for the determination of γ crystal orientation. Since the normals to (004) planes are parallel to macromolecular chains, the pole figures enabled the determination of the overall orientation of PVDF γ crystals for thermally treated multilayered PSF/PVDF film systems. Most of those γ crystalline lamellae are in-plane position which resulted from initial similar orientation of α crystals in as-extruded PSF/PVDF films. Further refinement of the texture occurs during isothermal recrystallization in conjunction with transformation of α to γ crystals and due to the interaction of PVDF with PSF/PVDF interface. The initial orientation of α crystals and resulted γ crystal orientation after α to γ transition are illustrated in Fig. 4.

Such in-plane orientation of polymer crystals with macromolecular chains perpendicular to the interface was reported previously by us for the system of nearly amorphous poly(ethylene-co-acrylic acid) (EAA) and crystalline polyethylene oxide (PEO) [53]. Such a possibility was also postulated by Ma, Hu, and Reiter defining such system as *crystals on sticky walls* [54].
